# Arithmetic Relationship between Fracture Load and Material Thickness of Resin-Based CAD-CAM Restorative Materials

**DOI:** 10.3390/polym14010058

**Published:** 2021-12-24

**Authors:** Tobias Graf, Josef Schweiger, Jan-Frederik Güth, Thomas Sciuk, Oliver Schubert, Kurt-Jürgen Erdelt

**Affiliations:** 1 Department of Prosthodontics, Center for Dentistry and Oral Medicine (Carolinum), Goethe-University Frankfurt am Main, 60596 Frankfurt am Main, Germany; gueth@med.uni-frankfurt.de; 2 Department of Prosthetic Dentistry, University Hospital, LMU Munich, 80336 Munich, Germany; josef.schweiger@med.uni-muenchen.de (J.S.); Oliver.Schubert@med.uni-muenchen.de (O.S.); Kurt.Erdelt@med.uni-muenchen.de (K.-J.E.); 3 Private Practice Thomas Sciuk, Prinzregentenstraße 8, 86150 Augsburg, Germany; thomas.sciuk@gmx.de

**Keywords:** CAD-CAM, digital workflow, fracture strength, fracture strength equation, hybrid materials, mathematical analysis, PMMA, polymer infiltrated ceramic network, resin nano ceramics

## Abstract

Data on the long-term behavior of computer-aided designed/computer-aided manufactured (CAD-CAM) resin-based composites are sparse. To achieve higher predictability on the mechanical behavior of these materials, the aim of the study was to establish a mathematical relationship between the material thickness of resin-based materials and their fracture load. The tested materials were Lava Ultimate (LU), Cerasmart (GC), Enamic (EN), and Telio CAD (TC). For this purpose, 60 specimens were prepared, each with five different material thicknesses between 0.4 mm and 1.6 mm (N = 60, n = 12). The fracture load of all specimens was determined using the biaxial flexural strength test (DIN EN ISO 6872). Regression curves were fitted to the results and their coefficient of determination (R^2^) was computed. Cubic regression curves showed the best R^2^ approximation (LU R^2^ = 0.947, GC R^2^ = 0.971, VE R^2^ = 0.981, TC R^2^ = 0.971) to the fracture load values. These findings imply that the fracture load of all tested resin-based materials has a cubic relationship to material thickness. By means of a cubic equation and material-specific fracture load coefficients, the fracture load can be calculated when material thickness is given. The approach enables a better predictability for resin-based restorations for the individual patient. Hence, the methodology might be reasonably applied to other restorative materials.

## 1. Introduction

Computer-aided design and computer-aided manufacturing (CAD-CAM) processes opened a broad range of ways to support or even replace conventional workflows in dentistry. In the field of fixed dental prosthodontics, CAD-CAM contributes to enhancing planning and efficient manufacture of dental prostheses [1,2].

Aside from all-ceramic restorations, resin-based CAD-CAM restorative materials present an interesting alternative to manufacture tooth colored, indirect single tooth restorations, e.g., crowns, partial crowns, or inlays [1,3]. The fabrication of these restorations is currently done almost entirely via additive or subtractive manufacturing processes based on CAD-CAM technologies [1,2]. The option of straightforward chairside restorations makes the use of these materials particularly appealing for dentistry and dental technology. Resin-based CAD-CAM materials can be used very efficiently within the digital workflow, allowing for the final restorations to be placed on the same day that the digital impression is taken [4], as these have to be polished after the milling or printing process. In contrast to ceramic restorations, no sintering processes are required to achieve the final strength [5,6]. These positive aspects resulted in a wide product range of alternative CAD-CAM-based materials [3]. The materials substantially differ regarding their composition and additives, such as quartz crystals, glasses, fibers, nanofillers, nanotubes, and hybrid materials, and are characterized by different physical and chemical properties [7].

While conventional dental resins comprise a matrix of monomers, Resin-Matrix-Ceramics (RMCs), also known as hybrid ceramics or nanoceramics, additionally contain significant proportions of ceramic components [3]. Resin-based composites include a polymer matrix and ceramic nanoparticles [8]. Depending on the manufacturer, they contain more than 70% weight/weight (% *w/w*) of nanoparticles made of different ceramics, such as zirconium oxide ceramics or glass ceramics based on barium or silicate [6]. So-called “polymer-infiltrated ceramic networks” (PICN) are composed of two interpenetrating networks of ceramic and polymer; this ceramic cluster, for example, consists of a feldspar ceramic [9].

A basic prerequisite of prosthodontic restorations is to remain in the oral cavity for as long as possible with no or minimum complications. Fractures or chipping are such frequent complications [10]. A sufficiently high mechanical stability of any restorative materials is crucial for long-term clinical success. Therefore, a vital parameter is the fracture load of a material, which depends on the individual geometry of an object, that is, in the dental context, the design of the restoration. Fracture load is measured in Newtons (N). An objective parameter indicating the resistance of a material is the flexural strength, determined for a restorative material applying a standardized method and thus serving as a reference for the stability of a material [11] which is measured in megapascals (MPa). The flexural strengths of resin-based and hybrid CAD-CAM restorative materials are significantly lower than those of zirconia or lithium disilicate ceramics [12]. Nevertheless, RMCs are approved for definitive restorations, depending on manufacturer and product.

An alteration in material thickness affects the stability of the restorative material. Therefore, it might be expected that increasing fracture load values of materials are associated with increasing layer thickness. This hypothesis has been confirmed in various in vitro studies by testing specimens with different layer thickness for their fracture load [13,14,15,16]. For instance, Nordahl et al. found that fracture load for monolithic zirconia ceramics and lithium disilicate ceramic decreased from thicker to thinner within the same material [15]. The same seems to apply for silicate ceramics and RMCs. For material thicknesses of 0.5, 1.0, and 1.5 mm, Zimmermann et al. observed statistically significant differences for the maximum fracture load depending on both the respective CAD-CAM material and layer thickness [16]. However, the question arises in everyday dental practice how the material thickness correlates with the fracture load.

Available clinical long-term data are very rare due to clinical implementation of these materials not occurring before the 2010s. Spitznagel et al. examined inlays and partial coverage restorations made from PICNs. For a total of 103 restoration in side teeth, survival rates of 97.4% for inlays and 95.6% for partial coverage restorations after three years were shown [17]. In another study by Spitznagel et al., 76 single crowns on full-coverage crown preparations with reduced thickness (1.0 to 1.5 mm) exhibited a survival rate of 94.7% after three years [18]. Endodontically treated side teeth in combination with CAD-CAM manufactured onlay restorations resulted in a survival rate of 97.0% after three years [19]. Therefore, PICN seems to be a suitable material option for posterior single-tooth restorations. However, extended clinical follow-up periods are needed to evaluate the long-term performance. For other RMCs, clinical long-term data are not available.

Against this background, it seems reasonable to adapt the stability of a restoration to the anticipated individual situation. The decision for or against the insertion of prosthetic restorations should be made based on objective parameters. The thickness of a material—determined by mechanical and biological factors—limits the selection of materials and the design of planned restorations to a sensible portfolio. Therefore, it seems reasonable to establish a correlation between fracture load and layer thickness using a mathematical equation with material-specific parameters. This allows for the predictive estimation of the individual conditions and means that further relevant knowledge might provide for the manufacture and clinical application of these restorative materials. Reliable estimation of the fracture load allows a minimally invasive preparation design to be realized [20]. Such an equation is therefore intended to create an innovative balance between the protection of valuable tooth structure and sufficiently high fracture load values of restorations.

For this purpose, three different CAD-CAM supported RMCs and one polymethylmethacrylate (PMMA) were investigated in the study. A linear correlation between material thickness and fracture load of each material was defined as the null hypothesis.

## 2. Material and Method

**Figure 1:**
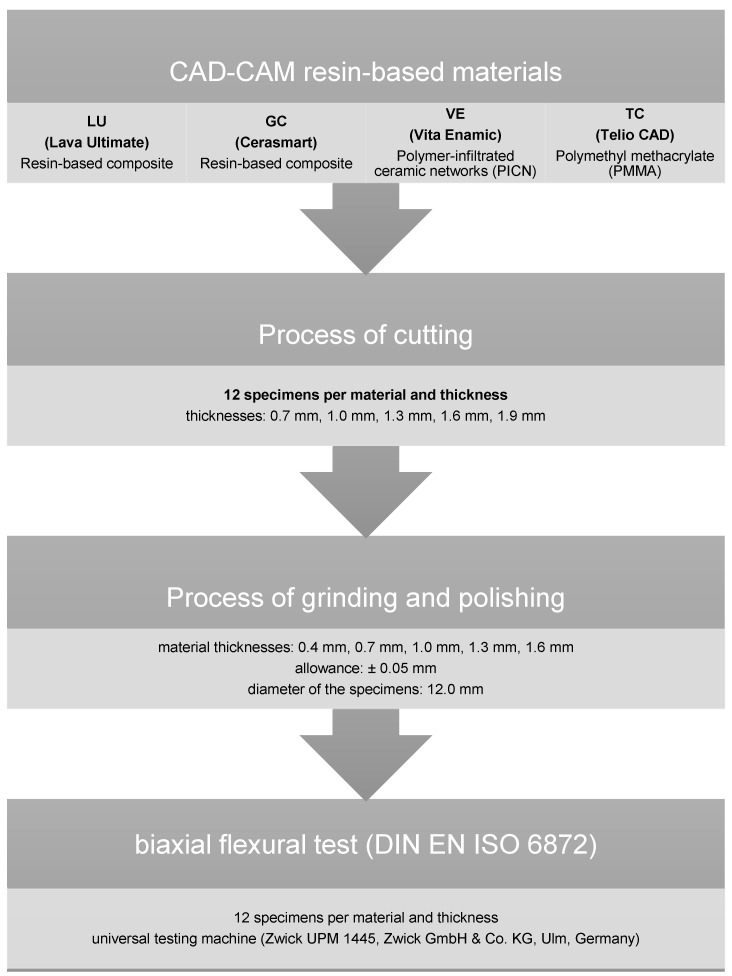
Experimental setup.

In the present study, the relationship between material thickness and fracture load was to be tested for Lava Ultimate A2-HT CAD/CAM (resin-based composite; 3M Espe AG, Seefeld, Germany) (LU), Cerasmart A2 HT 14 (resin-based composite; GC Europe N.V., Leuven, Belgium) (GC), Enamic 2 M2-HT EM 14 (PICN; VITA Zahnfabrik, Bad Säckingen, Germany) (VE), and Telio CAD LT A2/B40 L (polymethyl methacrylate; Ivoclar Vivadent AG, Schaan, Lichtenstein) (TC). Therefore, 60 test specimens (N = 60) with five different thicknesses (n = 12; 0.4 mm, 0.7 mm, 1.0 mm, 1.3 mm, and 1.6 mm) were prepared for each material (Figure 1).

First, cylinders with a diameter of 12.0 mm were designed using a CAD software (Solid Works 3D CAD Version 2020; Dassault Systèmes, Vélizy-Villacoublay, France) and then milled by the CAD-CAM machine Cerec inLab MC XL (Dentsply Sirona, York, PA, USA). The subtractively manufactured cylinders were subsequently cut to discs of 0.6 mm, 0.9 mm, 1.2 mm, 1.5 mm, and 1.8 mm thickness using a precision cutting machine (Secotom 50, Struers, Willich, Germany) with water cooling.

After cutting, the specimens were further processed using a polishing machine (Abramin; Struers) with water cooling to achieve the defined five different final material thickness. The maximum error tolerance in the manufacturing process was set to ±0.05 mm. The specimens were ground with diamond grinding wheels (MD Rondo; Struers) with 40 µm and 20 µm grain size. The surfaces were then polished using a polishing wheel (MD Largo; Struers) and diamond suspensions (DP-Suspension M; Struers) of different grain sizes (9 µm, 3 µm, 1 µm) without cooling. After the polishing process, all specimens were cleaned in the ultrasonic cleaner (Ultrasonic Cleaner T-14, L & R Manufacturing Company, Kearny, NJ, USA).

The fracture tests were performed using a universal testing machine (Zwick UPM 1445, Zwick GmbH & Co. KG, Ulm, Germany). The loading speed of the stainless-steel piston was 1.0 mm per minute. The test setup used based on the biaxial flexural strength test (DIN EN ISO 6872) [21]. The specimens were positioned on three supporting balls with a diameter of 3.2 ± 0.5 mm, which were angled at 120° from each other on a support disc with a diameter of 10.0 mm. The specimen was concentrically placed on these bearings and loaded at its center with a steel flat piston (diameter 1.7 mm).

Data analysis was performed using SPSS statistical software (Statistics 25.0, SPSS Inc., Stanford, CA, USA). All fracture load values were tested for normal distribution using the Kolmogorov–Smirnov test. Since a normal distribution could be assumed, the data were analyzed by parametric tests. Descriptive statistics were used to calculate the mean values including the standard deviations of the fracture load values. One-way ANOVA, followed by Scheffe’s post-hoc test, was used to determine the relation between fracture load values and material thickness. Regression analysis was used to determine the fit (coefficient of determination (R^2^)) for linear, quadratic, and cubic curve shapes.

## 3. Results

Table 1 lists the mean values including the standard deviations for the fracture loads. In addition, the flexural strength of the materials is given, which could be calculated according to the DIN standard EN ISO 6872.

The analysis of the statistical dependencies showed that all materials provided similar curve progressions. For every material, a similar statistical group behavior (“a”) was calculated for all thicknesses. Only GC displayed a different group behavior compared to the other materials in case of a thickness of 1.3 mm or more (Table 1).

**Figure 2:**
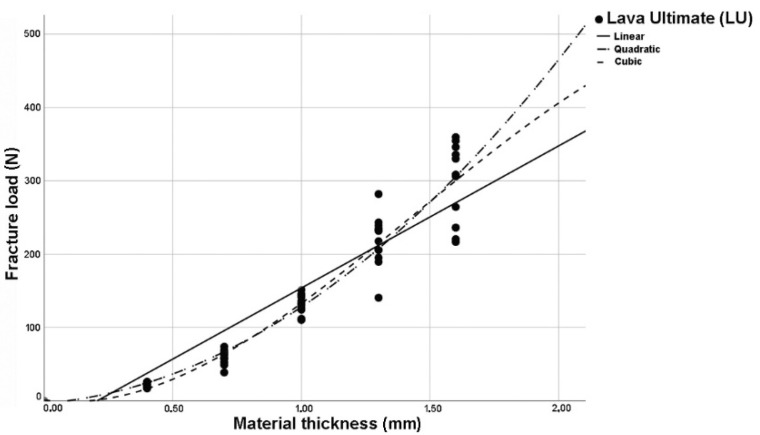
Plotted regression curves (linear, quadratic, and cubic) for Lava Ultimate (LU) with specimens of five thicknesses.

**Figure 3:**
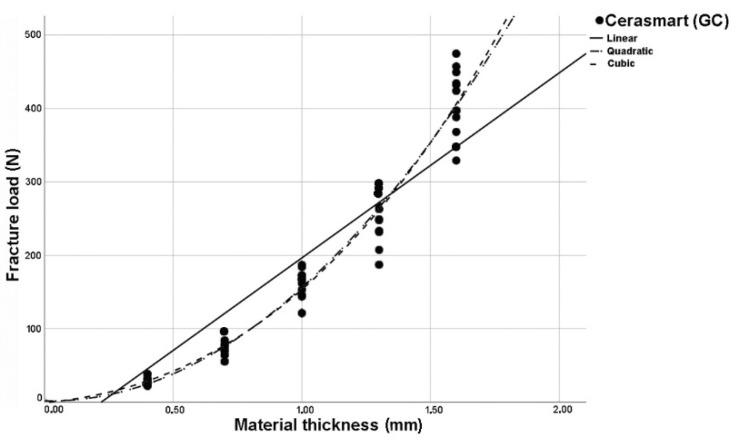
Plotted regression curves (linear, quadratic, and cubic) for Cerasmart (GC) with specimens of five thicknesses.

**Figure 4:**
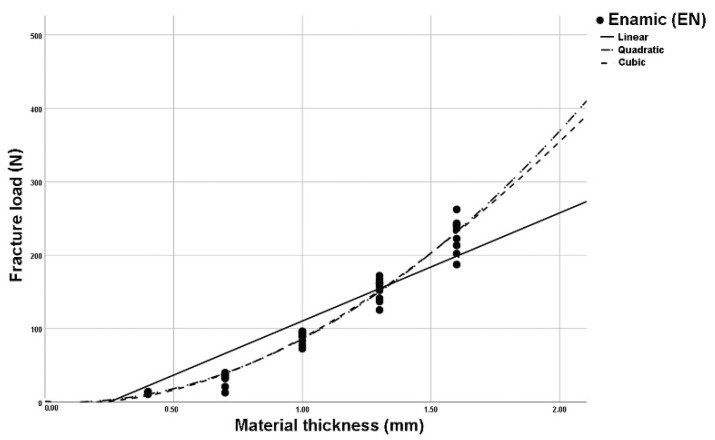
Plotted regression curves (linear, quadratic, and cubic) for Enamic (EN) with specimens of five thicknesses.

**Figure 5:**
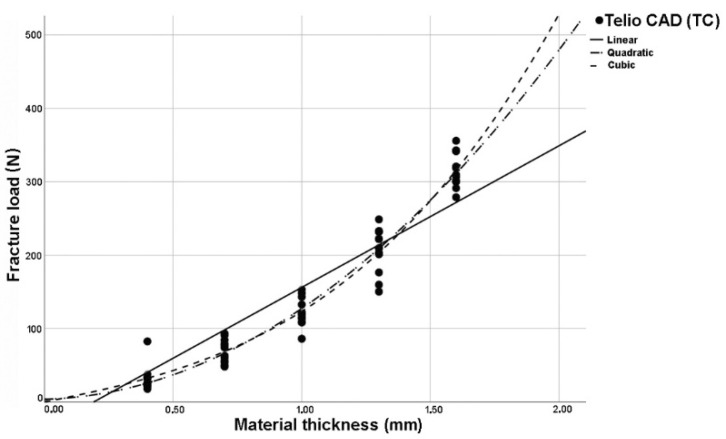
Plotted regression curves (linear, quadratic, and cubic) for Telio CAD (TC) with specimens of five thicknesses.

**Figure 6:**
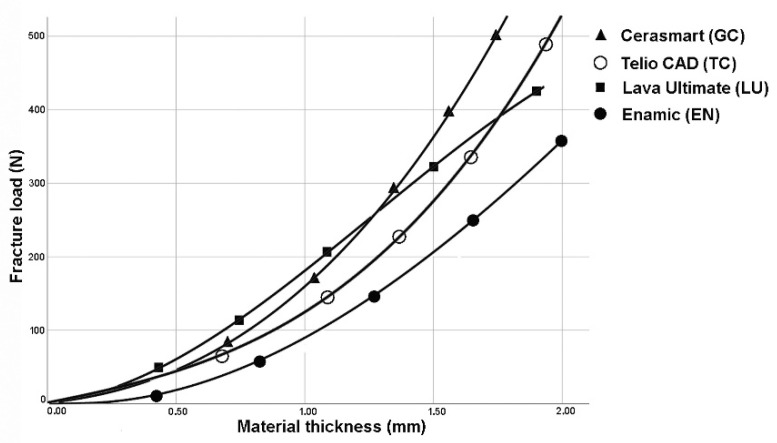
Cubic regression curves for all tested materials.

The analysis of regression curves for linear, quadratic, and cubic regression curves showed the best fit (R^2^) for cubic curves at LU and TC. GC and VE showed identical fit (R^2^) to quadratic and cubic curve shapes (Figure 2, Figure 3, Figure 4 and Figure 5). The closer R^2^ gets to a value of 1, the more exact the description of the translucency measurements points by the regression function. Thus, in case of all investigated materials, the cubic curve most precisely describes the fracture load curve between thicknesses of 0.4 mm to 1.6 mm (Table 2, Figure 6).

From these findings, a cubic functional equation could be developed which can be used to calculate the fracture load for any given material thickness in advance using material-specific coefficients (Table 3). This “Fracture Load Equation” is:

f(x) = b_0_ + b_1_∗x+ b_2_∗x^2^ + b_3_∗x^3^ (1)

f(x) = fracture load (N)

x = material thickness (mm)

b_0_, b_1_, b_2_, b_3_ = “Fracture Load Coefficients”.

## 4. Discussion

The null hypothesis must be rejected because cubic curves exhibit the best R^2^ approximation for all materials tested and therefore the relationship between fracture load and thickness is not linear but cubic.

The specimens’ surfaces and specimen diameters were prepared according to criteria of the DIN standard, so that the values of the specimens with a material thickness of 1.3 mm were also suitable for determining the flexural strength according to the requirements of the DIN standard [11]. Theoretically, the fracture load could be determined with a three-point bending test or a four-point bending test [11]. However, the biaxial flexural strength test holds substantial advantages concerning the simulation of multiaxial stress situations combined with generated stress peaks inside the test specimen, which more closely approximates the intraoral conditions when force is applied to restorations. Artificial aging prior to the strength load tests could be omitted because, according to Dikicier et al., increasing material thickness independently results in a higher fracture load [22]. Nevertheless, it must be mentioned in the context of the test specimen fabrication that the platelets were geometrically simple specimens which did not primarily represent the anatomic geometries of intraoral, prosthetic restorations (e.g., crowns, partial crowns, or inlays). However, the highly standardized experimental setup enables to determine and compare objective measurement parameters, which can serve as a basis for clinical practice.

To achieve best possible validity in studies it is crucial that the standard deviations of the measurement results are as low as possible, which can be accomplished by standardized manufacturing processes. All test specimens were subject to an identical workflow and manufacturing process and were loaded at the same points in the biaxial flexural strength test. Furthermore, the most accurate curve fit is achieved by a high number of interpolation points. A further support point was defined: for a material thickness of 0.0 mm, the fracture load was set to 0 N by definition. This resulted in a total of six supporting points, although only three would be necessary in theoretical analysis, providing more reliable results.

When analyzed for linear, quadratic, and cubic regression curves, cubic regression curves showed the best fit (R^2^) between thicknesses of 0.4 mm to 1.6 mm for all materials tested. Using the method of “least squares”, the deviation error of the measuring points to the regression curve can be minimized. Therefore, the curve may not exactly run through the zero point and little deviation can be observed.

Looking at the cubic regression curves, only small slopes of the graphs can initially be seen (Figure 6). The cubic equation implies that an increase of the material thickness has only a minor effect on the increase in fracture load values, especially in the case of thin material thicknesses. For practitioners, that relationship is of great relevance, since high stability of restorations is often required, but is only possible by a significant higher removal of valuable tooth structure during preparation. Since the masticatory forces vary depending on the intraoral position [23], it is of great benefit to be able calculate the fracture load as a function of the material thickness. Ferrario et al. reported a mean maximum masticatory force in the anterior region of up to 146 N, rising to 310 N in the posterior region [23]. In some cases of bruxism patients, values of up to 800 N can even be achieved in lateral tooth areas [24].

The usage of the tested materials would then be critical from the perspective of stability, since according to the calculations these values can only be achieved by very high removal rates (>2 mm) of valuable tooth structure. In this case, ceramic or metal-based restorations with higher flexural strength values would be required. Thus, there will be a significant increase in load strength, whereas “decent re-preparation”, in the sense of a further removal of tooth structure, does not appear to be sensible or effective for RMCs or PMMAs.

When assessing the fracture load values of prosthetic restorations, clinicians assume that there is a linear relationship between restoration thickness and fracture load. This, in turn, means that it might be supposed that doubling the crown thickness also means doubling the stability of the restoration and, the other way, that halving the crown thickness leads to half of the fracture load. This false assumption might lead to misjudgments regarding the long-term performance of the prosthodontic restorations. The estimation of non-linear relationships is difficult and might lead to severe errors in the assessment of the stability of restorations. The use of mathematical models might help to overcome these misconceptions in a direct and objective way.

Considering the work of Spitznagel et al., fractures in clinical use seem to be the predominant complication type in PICN based restorations, a subgroup of RMCs, as 3 out of 103 [17] and 4 out of 76 restorations [18] had to be replaced due to clinically unacceptable fractures within three years. Hence, a certain knowledge of the fracture load value of RMCs seems to be useful. Lu et al. found no fracture of the PICN-based restorations, but one debonding and one tooth fracture after three years of follow up were reported, implying that attention should also be paid to other parameters crucial for long-term success [19]. An early identification of a suitable material seems urgently necessary in order to minimize possible failure.

Regardless of thickness of restorations, the type of bonding or cementation can affect long-term performance. Several cements, such as zinc phosphate cement and glass ionomer cements, produce lower bond strengths, whereas bond strengths of self-etch and self-adhesive resin cements are significantly higher [25]. In general, adhesive luting composites are strongly recommended for restorative materials below flexural strength values of 350 MPa [26]. Rosentritt et al. confirmed this statement that a luting procedure should be preferred for RMCs in any case [27]. Adhesive bonding between tooth and material increases resistance and prevents debonding [26]. Nevertheless, according to the manufacturer’s guidelines, the indication of RMCs is exclusively for single-tooth restorations, independent of the material thicknesses used.

The calculation of the stability is possible by using the “Fracture Load Equation”. Table 4 lists fracture loads for given material thicknesses. Therefore, this table enables the forecast of the theoretical fracture load values in daily routine and might be a helpful material-specific decision guidance for assessing the stability regarding individual parameters. However, it seems much more practical to combine the “Fracture Load Equation” with the digital workflow by implementing the formula into the intraoral scanner or CAD software, which individually and automatically calculates the respective fracture load values during the design process and in the future, dependent on the planned restorative geometry, indication and patient´s individual bite forces. For this purpose, determining the material-specific parameters for other, preferably all, restorative materials would be useful to achieve universal practicability of this concept. However, the simple experimental setup should make this feasible for the manufacturers.

In addition to stability, esthetics play an important role in modern prosthodontics. The expectations of tooth-colored restorative materials grows steadily on the part of patients as well as clinicians. Therefore understanding, determining, and optimizing the optical characteristics of restorative materials is an important aspect to generate satisfying results. In this esthetic context, it is essential to consider that the translucency of the restoration changes, more precisely decreases due to a higher material thickness with tooth-colored, translucent materials [28,29,30]. Awad et al. found for RMCs and PMMAs that thickness is the major factor affecting the absolute translucency of adhesively luted restorations. However, surface roughness and pretreatment methods also influence the restorations’ translucency [28]. This often plays a key role, especially in the esthetic region. Moreover, the translucency of dental ceramics was significantly influenced by both material and thickness. The translucency of dental materials increased as the thickness decreased, but the amount of change was material-dependent [30]. Initial investigations here also showed that the optical behavior can be calculated using a mathematical equation. Ceramic materials, for example, exhibit a logarithmic relationship between layer thickness and light transmission [29].

Technological development of intraoral scanners now enables high reproducibility and accuracy of the intraoral situation on the one hand [31,32]. Secondly, color data can be recorded, compared, and processed further in manufacturing [33]. Moreover, internal tooth structures can be imaged in real time by near-infrared imaging (NIRI) technology, which supports caries detection by means of transillumination [34]. Further properties that are relevant for the long-term success of restorations should be determined in additional investigations. For example, the percentage of enamel, dentin, and build-up filling of the prepared tooth should be recorded in order to make a recommendation for the pretreatment steps during path of insertion.

In addition to fracture load and translucency, for example, thermal conductivity might be investigated as a relation to the material thickness of the restorative materials. The more physical properties are quantified or recorded, the more predictable restorations can be designed. Thus, more physical properties might be estimated in advance, i.e., during the CAD process, and reasonably implemented in CAD software which might furthermore install certain material-specific safety marks. Thus, more predictable, patient-centered, and individualized results might be achieved.

## 5. Conclusions

Using the biaxial flexural strength test, a mathematical relationship could be found between material thickness and fracture load of dental resin-based restorative materials (Lava Ultimate, Cerasmart, Enamic, and Telio CAD). Cubic regression curves showed the best R^2^ approximation (LU R^2^ = 0.947, GC R^2^ = 0.971, VE R^2^ = 0.981, TC R^2^ = 0.971), indicating a cubic relationship between fracture load and material thickness. By using the material-specific parameters which determined in this study, a cubic “Fracture Load Equation” can be formulated as follows: f (x) = b_0_ + b_1_∗x + b_2_∗x^2^ + b_3_∗x^3^. Thus, the calculation of the fracture load is possible for any given layer thickness. This helps to enable a better patient- and situation-specific estimation of the fracture load in advance, depending on the geometry of designed prosthetic restorations. The findings result in a significantly higher predictability for the final restoration and is expected to have a positive impact on the clinical long-term performance. Since resin-based materials are almost completely manufactured using CAD-CAM processes, it seems desirable to implement the “Fracture Load Equation” into CAD software. In future, material-specific safety parameters might be automatically regarded during manufacturing process. Due to the simple experimental setup, this methodology might be applied to find material-specific “Fracture Load Coefficients” for other dental restorative materials.

## Figures and Tables

**Table 1:** Descriptive statistics of fracture load values including mean values, standard deviations (SD), and statistical significance (Scheffe’s post-hoc test). Same letters indicate no statistically significant differences concerning fracture load values (a, b).

	Material Thickness					Flexural Strength
	0.4 mm	0.7 mm	1.0 mm	1.3 mm	1.6 mm	
material	mean ± SD (N)	mean ± SD (N)	mean ± SD (N)	mean ± SD (N)	mean ± SD (N)	mean ± SD (MPa)
Lava Ultimate (LU)	21.3 ± 3.1 “a”	58.9 ± 9.3 “a”	132.1 ± 12.8 “a”	218.2 ± 35.0 “a”	289.8 ± 35.0 “a”	223.3 ± 20.0
GC Smart (GC)	27.7 ± 47.0 “a”	74.6 ± 10.2 “a”	162.1 ± 18.5 “a”	254.3 ± 35.1 “b”	408.2 ± 44.9 “b”	276.3 ± 41.0
Vita Enamic (VE)	13.3 ± 1.0 “a”	32,6 ± 7.9 “a”	88.8 ± 7.3 “a”	153.4 ± 14.7 “a”	230.0 ± 20.5 “a”	132.4 ± 10.2
Telio CAD (TC)	32.3 ± 16.9 “a”	69.2 ± 16.0 “a”	122.5 ± 19.1 “a”	204.0 ± 29.5 “a”	315.5 ± 22.5 “a”	187.8 ± 27.6

**Table 2:** R^2^ values of the linear, quadratic, and cubic curves for all tested materials (* fracture load values that fit the curves best).

Material	Linear	Quadratic	Cubic
Lava Ultimate (LU)	0.886	0.944	0.947 *
GC Smart (GC)	0.886	0.971 *	0.971 *
Enamic (VE)	0.888	0.981 *	0.981 *
Telio CAD (TC)	0.896	0.969	0.971 *

**Table 3:** Material-specific “Fracture Load Coefficients” ‘b_0_’, ‘b_1_’, ‘b_2_’, and ‘b_3_’ with mean values and standard deviations.

Material	Cubic “Fracture Load Coefficients”			
	b_0_	b_1_	b_2_	b_3_
Lava Ultimate (LU)	1.1 ± 0.5	−45.1 ± 44.6	230.0 ± 70.0	−53.1 ± 28.8
GC Smart (GC)	−0.57 ± 7.2	31.0 ± 42.5	95.8 ± 66.7	27.3 ± 27.5
Enamic (VE)	1.0 ± 3.5	−30.7 ± 20.1	129.7 ± 31.3	−13.0 ± 12.9
Telio CAD (TC)	0.0 ± 5.5	69.9 ± 51.3	9.8 ± 21.2	43.7 ± 5.5

**Table 4:** Calculated fracture load values (in N) for Cerasmart (GC), Lava Ultimate (LU), Enamic (VE), and Telio CAD (TC) using the “Fracture Load Equation” for given thicknesses.

Material-Dependent Calculated Fracture Loads	Material Thickness (in mm)																	
	0.3	0.4	0.5	0.6	0.7	0.8	0.9	1.0	1.1	1.2	1.3	1.4	1.5	1.6	1.7	1.8	1.9	2.0
Lava Ultimate (LU) (in N)	6.9	16.5	29.4	45.4	64.1	85.1	108.1	132.9	159.2	186.5	214.5	243.1	271.8	300.3	328.2	355.4	381.5	406.1
GC Smart (GC) (in N)	18.1	28.9	42.3	58.4	77.4	99.5	124.8	153.5	185.8	221.8	261.6	305.5	353.6	406.1	463.2	524.9	591.5	663.1
Enamic (VE) (in N)	3.2	8.7	16.5	26.5	38.7	52.9	69.0	87.1	107.0	128.6	151.8	176.7	203.0	230.8	259.9	290.3	321.9	354.6
Telio CAD (TC) (in N)	23.0	32.3	42.9	54.9	68.7	84.5	102.7	123.4	146.8	173.4	203.3	236.8	274.2	315.7	361.6	412.1	467.6	528.2

## Data Availability

Not applicable.
